# Supplementary material for: MIP-1α Expression Induced by Co-Stimulation of Human Monocytic Cells with Palmitate and TNF-α Involves the TLR4-IRF3 Pathway and Is Amplified by Oxidative Stress
Source: Cells. 2020 Jul 29;9(8):1799. doi: 10.3390/cells9081799 (PMC7465096; doi:10.3390/cells9081799)
Supplement: Supplementary file 1 [file cells-09-01799-s001.pdf]

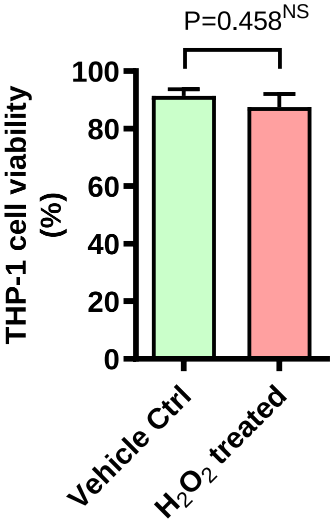

Figure S1: Viability of THP-1 cells treated with H_2O_2. THP-1 cells were plated (1×10^6 cells/mL/well) in triplicate wells of 12-well plates and incubated with H_2O_2 (200µM) at 37°C for 10h for induction of oxidative stress., while the cells in control wells were incubated with vehicle (1% BSA) only. Cell viability (%) was determined by trypan blue dye exclusion test. The representative data (mean±SEM) from three independent determinations with similar results show that cell viability differed non-significantly between H_2O_2 treatment and control (P=0.458)

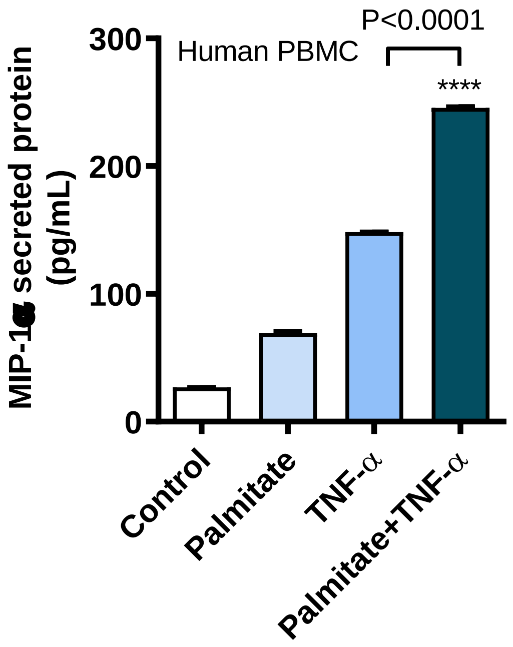

Figure S2. Palmitate and TNF-*α* co-induce MIP-1*α* expression in the human peripheral blood mononuclear cells (PBMC). Human PBMC were isolated from the freshly collected peripheral blood samples from 3 healthy donors, cells were plated (1×10^6 cells/mL/well) in triplicate wells of 12-well plates and stimulated with palmitate (200 μM) and/or TNF-*α* (10 ng/ml) or 0.1% BSA (Control) and incubated at 37°C for 24h as described in Materials and Methods. MIP-1*α* secreted protein was measured in cell supernatants using commercial ELISA kit and following the manufacturer's instructions. The representative data (mean±SEM) from three independent determinations with similar results show significantly elevated expression of MIP-1*α* secreted protein in cells that were co-stimulated with palmitate and TNF-*α* (245.70±1.21 pg/mL) as compared to stimulation with TNF-*α* alone (148.50±0.41 pg/mL) ($P<0.0001$).

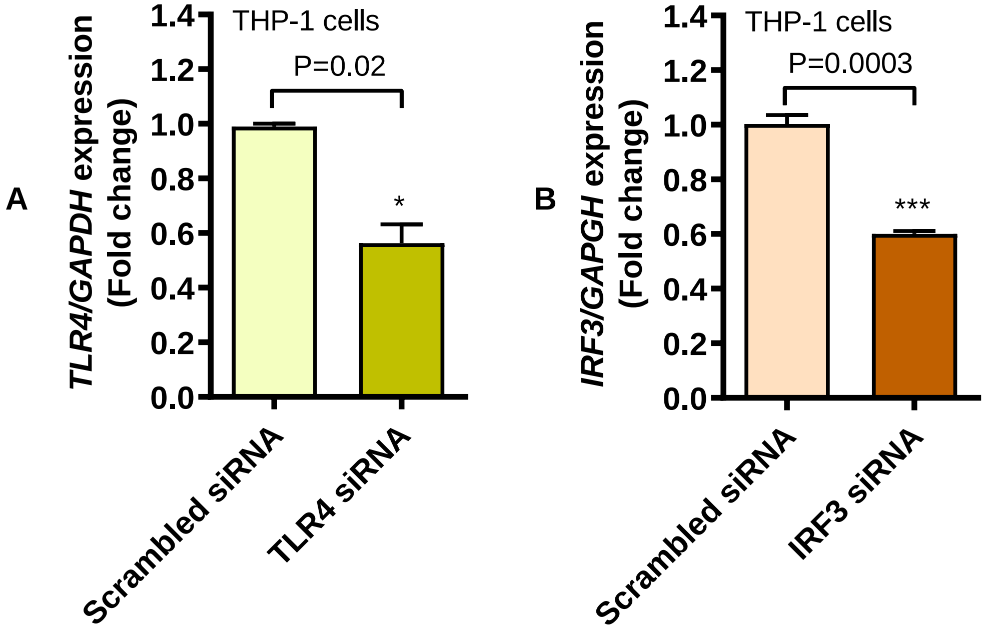

Figure S3. Genetic ablation of TLR4 and IRF3 in THP-1 monocytic cells. THP-1 monocytic cells were transfected separately with TLR4-/IRF3-specific siRNAs (30 nM each) or scrambled siRNA (30 nM) and pmaxGFP (0.5μg) using Amaxa Electroporation System as described in Materials and Methods. At 36h post-transfection, THP-1 cells (10^6 cells/mL/well) were treated in triplicate wells with palmitate (200 μM) and/or TNF-α (10 ng/ml) or 0.1% BSA and incubated at 37°C for 24h. Cells were harvested for total RNA extraction and the efficiency of siRNA-mediated target gene suppression was assessed using real-time qRT-PCR. The representative data (mean±SEM) from three independent determinations with similar results show significant suppression of (**A**) TLR4 (a reduction from 0.99±0.01 folds to 0.56±0.06 folds; P=0.02) and (**B**) IRF3 expression (a reduction from 0.99±0.03 folds to 0.60±0.01 folds; P=0.0003) in target gene specific siRNA transfected cells as compared to scrambled siRNA transfected controls.

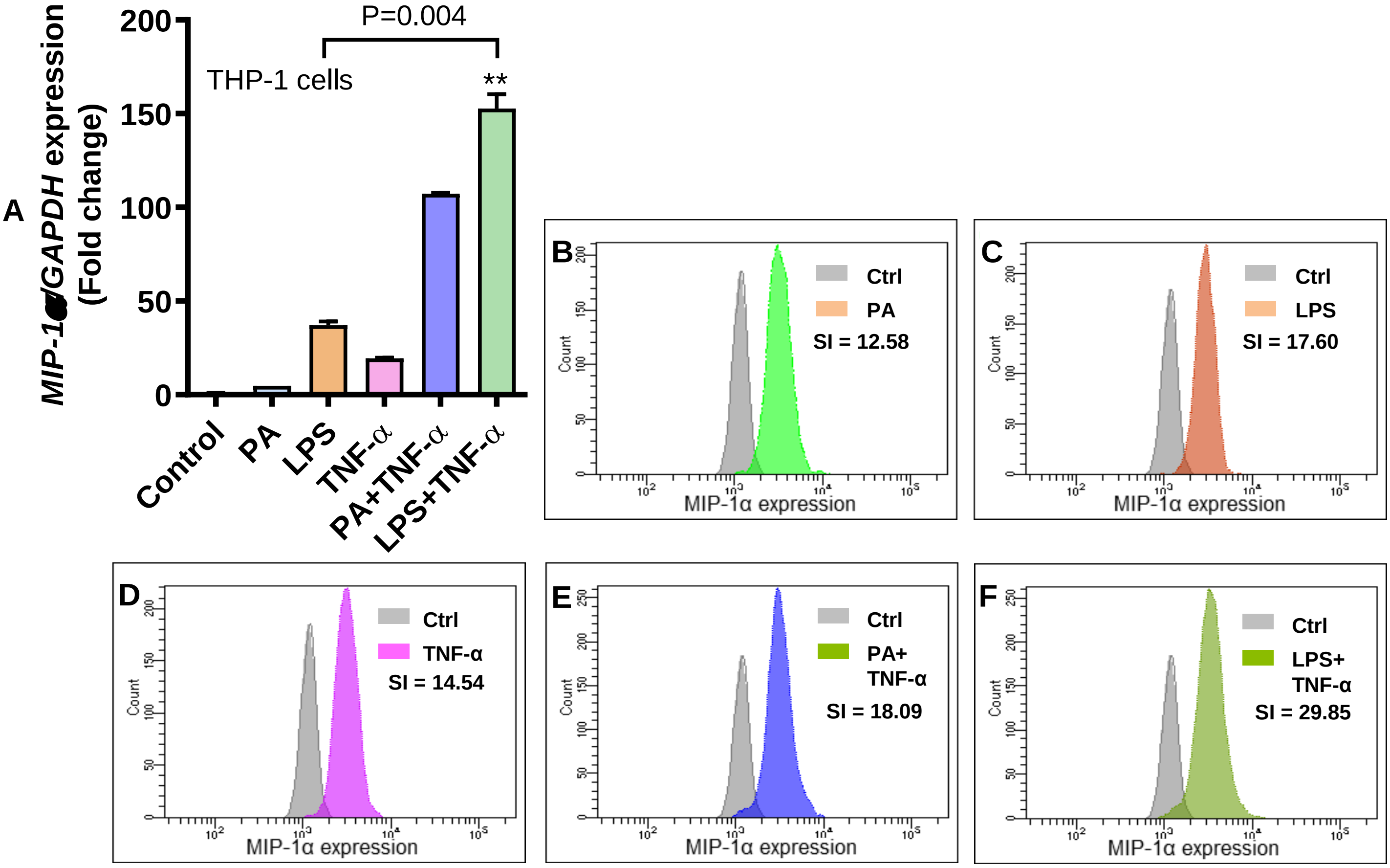

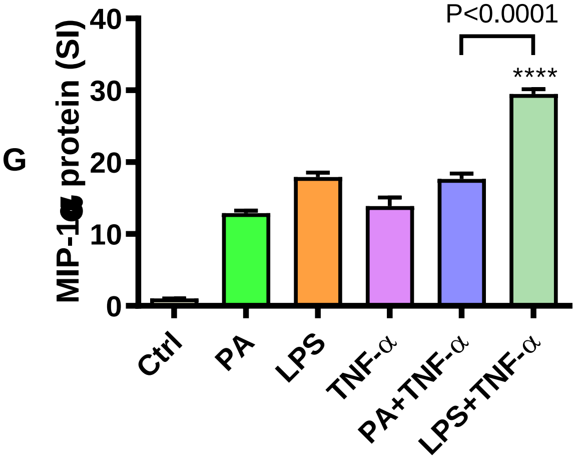

Figure S4. MIP-1*α* induction in THP-1 monocytic cells by canonical and non-canonical TLR4 activators. THP-1 cells were plated (1×10^6 cells/mL/well) in triplicate wells of 12-well plates and stimulated with 200μM palmitate (PA; a non-canonical agonist), 10μg/mL LPS (a canonical activator), 10μg/mL TNF-*α*, PA+TNF-*α*, and LPS+TNF-*α* while controls were treated with 0.1% BSA, and the cells were incubated at 37°C for 24h. Total RNA was collected for assessing MIP-1*α* gene expression and cells were stained for determining intracellular MIP-1*α* protein expression as described in Materials and Methods. The representative data (mean±SEM) from three independent determinations with similar results show (**A**) higher MIP-1*α* gene expression induced by LPS+TNF-*α* than by PA+TNF-*α* (P=0.004). (**B-F**) Representative histograms show MIP-1*α* protein expression. (**G**) MIP-1*α* protein expression induced by LPS+TNF-*α* was higher than that induced by PA+TNF-*α* (P<0.0001).

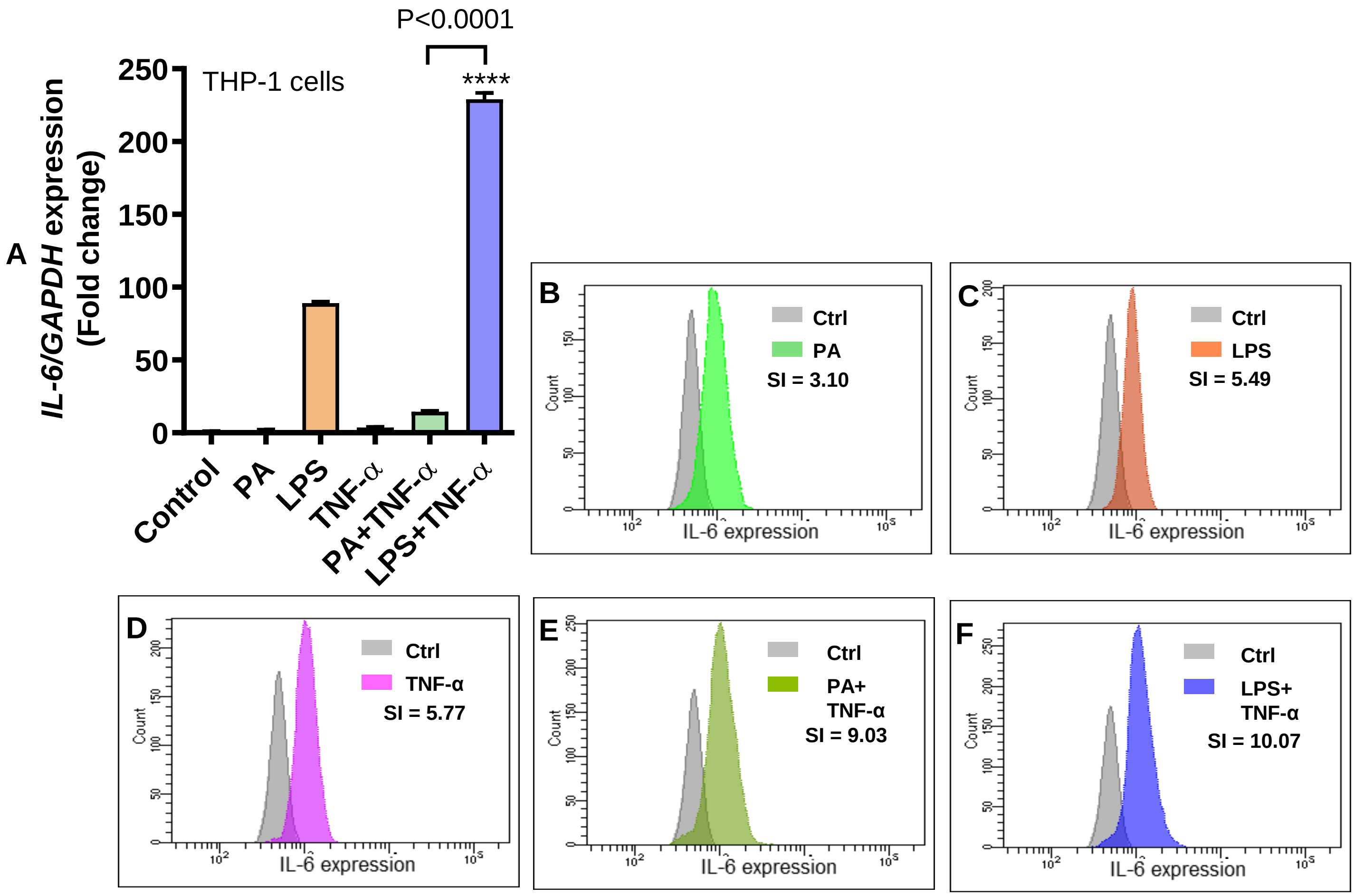

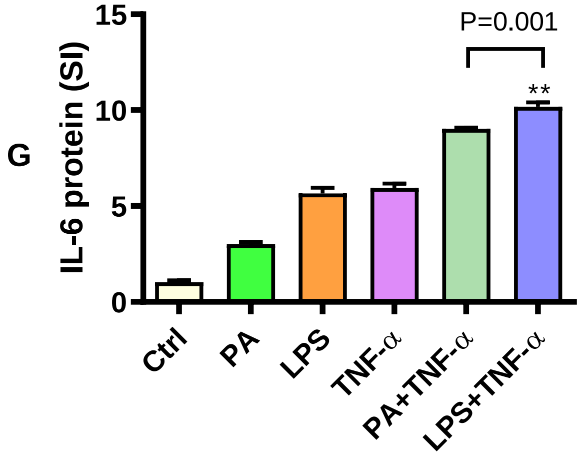

Figure S5. Comparative IL-6 induction by LPS and palmitate, in combination with TNF-α, in THP-1 monocytic cells. THP-1 cells were plated (1×10^6 cells/mL/well) in triplicate wells of 12-well plates and stimulated with 200μM palmitate (PA; a non-canonical agonist), 10μg/mL LPS (a canonical activator), 10μg/mL TNF-α, PA+TNF-α, and LPS+TNF-α, while controls were treated with 0.1% BSA, and the cells were incubated at 37°C for 24h. Total RNA was collected for assessing IL-6 gene expression and cells were stained to determine intracellular IL-6 protein expression. The representative data (mean±SEM) from three independent determinations with similar results show (**A**) higher IL-6 gene expression induced by LPS+TNF-α than by PA+TNF-α (P=0.004). (**B-F**) Representative histograms show IL-6 protein expression. (**G**) IL-6 protein expression induced by LPS+TNF-α was higher than that induced by PA+TNF-α (P=0.001).
